# Supplementary material for: Machine learning and natural language processing to assess the emotional impact of influencers’ mental health content on Instagram
Source: PeerJ Comput Sci. 2024 Sep 19;10:e2251. doi: 10.7717/peerj-cs.2251 (PMC11419624; doi:10.7717/peerj-cs.2251)
Supplement: Supplemental Information 1 [file peerj-cs-10-2251-s001.docx]

**Table 1:**

**Selected Instagram posts related to mental health.**

| Influencer | Followers (million) | Instagram Post | Number of responses |
| --- | --- | --- | --- |
| Laura Escanes | 1.9 | https://www.instagram.com/p/CSzr2hnDICO/ | 1485 |
| Dulceida | 3.3 | <https://www.instagram.com/p/CW_ZiztIeDx/> | 3507 |
| Dulceida | 3.3 | <https://www.instagram.com/reel/CS4lWBEi_zk> | 3461 |
| María Pombo | 0.744 | <https://www.instagram.com/tv/CKysupPAXF7> | 7093 |
| Elisabeth Cardi | 0.1 | <https://www.instagram.com/p/Ci-yET2sDj3/> | 33 |
| María rivers | 1.9 | https://www.instagram.com/reel/CjqqwRkIGpn | 201 |
| Susana Bicho | 1 | https://www.instagram.com/tv/CXQ7ytHqMXP | 64 |
| Tamara Gorro | 2 | https://www.instagram.com/tv/CbKt5p-lSb2 | 665 |
| Tamara Gorro | 2 | <https://www.instagram.com/p/CclZgKetEFw/> | 1950 |
| Tamara Gorro | 2 | <https://www.instagram.com/p/CYmBCryt8wQ/?hl=es> | 2070 |
| Tania Llasera | 0.793 | <https://www.instagram.com/p/CGmgxXVp6dH> | 622 |

**Table order:**

Table 1 appears first in the text, and first cited.
