# Supplementary material for: Machine learning and natural language processing to assess the emotional impact of influencers’ mental health content on Instagram
Source: PeerJ Comput Sci. 2024 Sep 19;10:e2251. doi: 10.7717/peerj-cs.2251 (PMC11419624; doi:10.7717/peerj-cs.2251)
Supplement: Supplemental Information 2 [file peerj-cs-10-2251-s002.docx]

**Table 2:**

**Optimisation of the hyperparameter number of features.**

| Max_features | Accuracy (%) |
| --- | --- |
| Log2 | 44 |
| Sqrt | 46 |

**Table orders:**

Table 2 appears second, and the next cited after Table 1
