# Supplementary material for: Machine learning and natural language processing to assess the emotional impact of influencers’ mental health content on Instagram
Source: PeerJ Comput Sci. 2024 Sep 19;10:e2251. doi: 10.7717/peerj-cs.2251 (PMC11419624; doi:10.7717/peerj-cs.2251)
Supplement: Supplemental Information 3 [file peerj-cs-10-2251-s003.docx]

**Table 3:**

**Optimisation of the number of trees hyperparameter.**

| Number trees | Accuracy (%) |
| --- | --- |
| 10 | 45 |
| 100 | 46 |
| 300 | 47 |
| 500 | 48 |
| 700 | 47 |
| 1000 | 46 |

Table order:

Table 3 appears second, and the next cited after Table 2
