# Supplementary material for: Machine learning and natural language processing to assess the emotional impact of influencers’ mental health content on Instagram
Source: PeerJ Comput Sci. 2024 Sep 19;10:e2251. doi: 10.7717/peerj-cs.2251 (PMC11419624; doi:10.7717/peerj-cs.2251)
Supplement: Supplemental Information 4 [file peerj-cs-10-2251-s004.docx]

**Table 4:**

**RF results for accuracy, recall and F1-score metrics considering all emotions.**

| Emotions | Precision (%) | Recall (%) | F1-score (%) |
| --- | --- | --- | --- |
| Love/Admiration | 48 | 56 | 51 |
| Gratitude | 87 | 54 | 67 |
| Sadness | 40 | 10 | 16 |
| Anger/Contempt/Mockery | 51 | 25 | 34 |
| Comprehension/Empathy/Identification | 45 | 70 | 55 |
| Neutral | 37 | 27 | 31 |
| Global (weighted avg) | 50 | - | 47 |

**Table orders:**

Table 4 appears second, and the next cited after Table 3
