# Supplementary material for: Machine learning and natural language processing to assess the emotional impact of influencers’ mental health content on Instagram
Source: PeerJ Comput Sci. 2024 Sep 19;10:e2251. doi: 10.7717/peerj-cs.2251 (PMC11419624; doi:10.7717/peerj-cs.2251)
Supplement: Supplemental Information 5 [file peerj-cs-10-2251-s005.docx]

**Table 5:**

**Summary of tests performed to assess hyperparameters, number of neurons and filters.**

| Number filters Convolutional layer | Number neurons LSMT layer | Dropout parameter | Recurrent parameter | Kernel size | Cross-validation Accuracy (%) |
| --- | --- | --- | --- | --- | --- |
| 192 | 256 | 0.2 | 0.3 | 8 | 67.83 |
| 192 | 128 | 0.2 | 0.3 | 8 | 68.56 |
| 192 | 96 | 0.2 | 0.3 | 8 | 68.41 |
| 192 | 64 | 0.2 | 0.3 | 8 | 69.14 |
| 180 | 256 | 0.2 | 0.3 | 8 | 69.14 |
| 180 | 96 | 0.2 | 0.3 | 8 | 72.05 |
| 160 | 128 | 0.2 | 0.3 | 8 | 67.25 |
| 160 | 64 | 0.2 | 0.3 | 8 | 67.25 |
| 150 | 256 | 0.2 | 0.3 | 8 | 66.67 |
| 128 | 128 | 0.2 | 0.3 | 8 | 68.41 |
| 128 | 64 | 0.2 | 0.3 | 8 | 69.29 |
| 96 | 64 | 0.2 | 0.3 | 8 | 68.41 |
| 180 | 128 | 0.2 | 0.3 | 8 | 69.14 |

**Table orders:**

Table 5 appears second, and the next cited after Table 4
