# Supplementary material for: Machine learning and natural language processing to assess the emotional impact of influencers’ mental health content on Instagram
Source: PeerJ Comput Sci. 2024 Sep 19;10:e2251. doi: 10.7717/peerj-cs.2251 (PMC11419624; doi:10.7717/peerj-cs.2251)
Supplement: Supplemental Information 6 [file peerj-cs-10-2251-s006.docx]

**Table 6:**

**Results obtained for dropout rate and recurrent dropout variation.**

| Number filters Convolutional layer | Number neurons LSMT layer | Dropout parameter | Recurrent parameter | Kernel size | Cross-Validation Accuracy (%) |
| --- | --- | --- | --- | --- | --- |
| 192 | 96 | 0.2 | 0.3 | 8 | 72.05 |
| 192 | 96 | 0.3 | 0.3 | 8 | 65.50 |
| 192 | 96 | 0.4 | 0.3 | 8 | 70.16 |
| 192 | 96 | 0.5 | 0.3 | 8 | 69.87 |
| 192 | 96 | 0.6 | 0.3 | 8 | 69.29 |
| 192 | 96 | 0.7 | 0.3 | 8 | 69.14 |
| 192 | 96 | 0.8 | 0.3 | 8 | 65.07 |
| 192 | 96 | 0.2 | 0.4 | 8 | 67.69 |
| 192 | 96 | 0.2 | 0.5 | 8 | 68.27 |
| 192 | 96 | 0.2 | 0.6 | 8 | 68.41 |
| 192 | 96 | 0.2 | 0.7 | 8 | 69.29 |
| 192 | 96 | 0.2 | 0.8 | 8 | 68.85 |

**Table orders:**

Table 6 appears second, and the next cited after Table 5
