# Supplementary material for: Machine learning and natural language processing to assess the emotional impact of influencers’ mental health content on Instagram
Source: PeerJ Comput Sci. 2024 Sep 19;10:e2251. doi: 10.7717/peerj-cs.2251 (PMC11419624; doi:10.7717/peerj-cs.2251)
Supplement: Supplemental Information 7 [file peerj-cs-10-2251-s007.docx]

**Table 7:**

**Results obtained for different values of learning rate.**

| Learning rate | Cross-Validation Accuracy (%) |
| --- | --- |
| 0.006 | 66.81 |
| 0.008 | 70.31 |
| 0.01 | 72.05 |
| 0.02 | 67.83 |
| 0.04 | 38.43 |

**Table orders:**

Table 7 appears second, and the next cited after Table 6
