# Supplementary material for: Machine learning and natural language processing to assess the emotional impact of influencers’ mental health content on Instagram
Source: PeerJ Comput Sci. 2024 Sep 19;10:e2251. doi: 10.7717/peerj-cs.2251 (PMC11419624; doi:10.7717/peerj-cs.2251)
Supplement: Supplemental Information 8 [file peerj-cs-10-2251-s008.docx]

**Table 8:**

**Results obtained for different vocabulary sizes.**

| Vocabulary size | Cross-Validation  Accuracy (%) |
| --- | --- |
| 2457 | 69.14 |
| 2357 | 68.56 |
| 2257 | 69.58 |
| 2157 | 70.60 |
| 1957 | 72.5 |
| 1857 | 70 |

**Table orders:**

Table 8 appears second, and the next cited after Table 7
