# Supplementary material for: Machine learning and natural language processing to assess the emotional impact of influencers’ mental health content on Instagram
Source: PeerJ Comput Sci. 2024 Sep 19;10:e2251. doi: 10.7717/peerj-cs.2251 (PMC11419624; doi:10.7717/peerj-cs.2251)
Supplement: Supplemental Information 9 [file peerj-cs-10-2251-s009.docx]

**Table 9:**

**Summary of the hybrid deep learning model results for precision, recall and F1-score.**

| Emotions | Precision (%) | Recall (%) | F1-score (%) |
| --- | --- | --- | --- |
| Love/Admiration | 77 | 81 | 79 |
| Comprehension/Empathy/Identification | 66 | 77 | 71 |
| Sadness | 60 | 51 | 55 |
| Gratitude | 85 | 85 | 65 |
| Anger/Contempt/Mockery | 84 | 68 | 75 |
| Neutral | 60 | 51 | 55 |
| Global (weighted avg) | 73 |  | 72 |

**Table orders:**

Table 9 appears second, and the next cited after Table 8
