# Supplementary material for: Machine learning and natural language processing to assess the emotional impact of influencers’ mental health content on Instagram
Source: PeerJ Comput Sci. 2024 Sep 19;10:e2251. doi: 10.7717/peerj-cs.2251 (PMC11419624; doi:10.7717/peerj-cs.2251)
Supplement: Supplemental Information 10 [file peerj-cs-10-2251-s010.docx]

**Table 10:**

**Summary of Danevi and RoBERTuito results for precision, recall and F1-score metrics.**

| Emotion | Metric | Danevi (%) | RoBERTuito (%) |
| --- | --- | --- | --- |
| Love/Admiration | Precision | 90.6 | 93.1 |
|  | Recall | 92.7 | 95 |
|  | F1-score | 91.6 | 94 |
| Gratitude | Precision | 94.1 | 90.8 |
|  | Recall | 89 | 92.1 |
|  | F1-score | 91.4 | 91.3 |
| Sadness | Precision | 81.4 | 83.7 |
|  | Recall | 79.4 | 87.8 |
|  | F1-score | 79.3 | 85.1 |
| Anger/Contempt/Mockery | Precision | 84.7 | 91 |
|  | Recall | 85.8 | 93.2 |
|  | F1-score | 84.9 | 92 |
| Comprehension/Empathy/Identification | Precision | 84.6 | 89.9 |
|  | Recall | 88.6 | 88.5 |
|  | F1-score | 86.3 | 89.1 |
| Neutral | Precision | 70.1 | 79.5 |
|  | Recall | 52.6 | 70.9 |
|  | F1-score | 57.4 | 65 |
| Global | Accuracy | 86 | 90 |

**Table orders:**

Table 10 appears second, and the next cited after Table 9
