# Supplementary material for: Machine learning and natural language processing to assess the emotional impact of influencers’ mental health content on Instagram
Source: PeerJ Comput Sci. 2024 Sep 19;10:e2251. doi: 10.7717/peerj-cs.2251 (PMC11419624; doi:10.7717/peerj-cs.2251)
Supplement: Supplemental Information 11 [file peerj-cs-10-2251-s011.docx]

**Table 11:**

**Comparison of all algorithms for the accuracy metric.**

| RoBERTuito (%) | Danevi (%) | Deep Learning (%) | RF (%) |
| --- | --- | --- | --- |
| 90 | 86 | 72.5 | 48 |

**Table orders:**

Table 11 appears second, and the next cited after Table 10
