# Supplementary material for: Machine learning and natural language processing to assess the emotional impact of influencers’ mental health content on Instagram
Source: PeerJ Comput Sci. 2024 Sep 19;10:e2251. doi: 10.7717/peerj-cs.2251 (PMC11419624; doi:10.7717/peerj-cs.2251)
Supplement: Supplemental Information 12 [file peerj-cs-10-2251-s012.docx]

**Table 12:**

**Comparison of all algorithms for accuracy metrics, recall and F1 score considering the emotional classes (six emotions plus a Neutral class).**

| **Emotion** | **Metric** | **Danevi** (%) | **RoBERTuito** (%) | **Deep Learning** (%) | **RF** (%) |
| --- | --- | --- | --- | --- | --- |
| Love/Admiration | Precision | 90.6 | 93.1 | 77 | 48 |
|  | Recall | 92.7 | 95 | 81 | 56 |
|  | F1-score | 91.6 | 94 | 79 | 51 |
| Gratitude | Precision | 94.1 | 90.8 | 85 | 87 |
|  | Recall | 89 | 92.1 | 85 | 54 |
|  | F1-score | 91.4 | 91.3 | 65 | 67 |
| Sadness | Precision | 81.4 | 83.7 | 60 | 40 |
|  | Recall | 79.4 | 87.8 | 51 | 10 |
|  | F1-score | 79.3 | 85.1 | 55 | 16 |
| Anger/Contempt/Mockery | Precision | 84.7 | 91 | 84 | 51 |
|  | Recall | 85.8 | 93.2 | 68 | 25 |
|  | F1-score | 84.9 | 92 | 75 | 35 |
| Comprehension/Empathy  /Identification | Precision | 84.6 | 89.9 | 66 | 45 |
|  | Recall | 88.6 | 88.5 | 77 | 70 |
|  | F1-score | 86.3 | 89.1 | 71 | 55 |
| Neutral | Precision | 70.1 | 79.5 | 60 | 37 |
|  | Recall | 52.6 | 70.9 | 51 | 27 |
|  | F1-score | 57.4 | 65 | 55 | 31 |

**Table orders:**

Table 12 appears second, and the next cited after Table 11
