# Supplementary figures and images for: Machine learning and natural language processing to assess the emotional impact of influencers’ mental health content on Instagram
Source: PeerJ Comput Sci. 2024 Sep 19;10:e2251. doi: 10.7717/peerj-cs.2251 (PMC11419624; doi:10.7717/peerj-cs.2251)

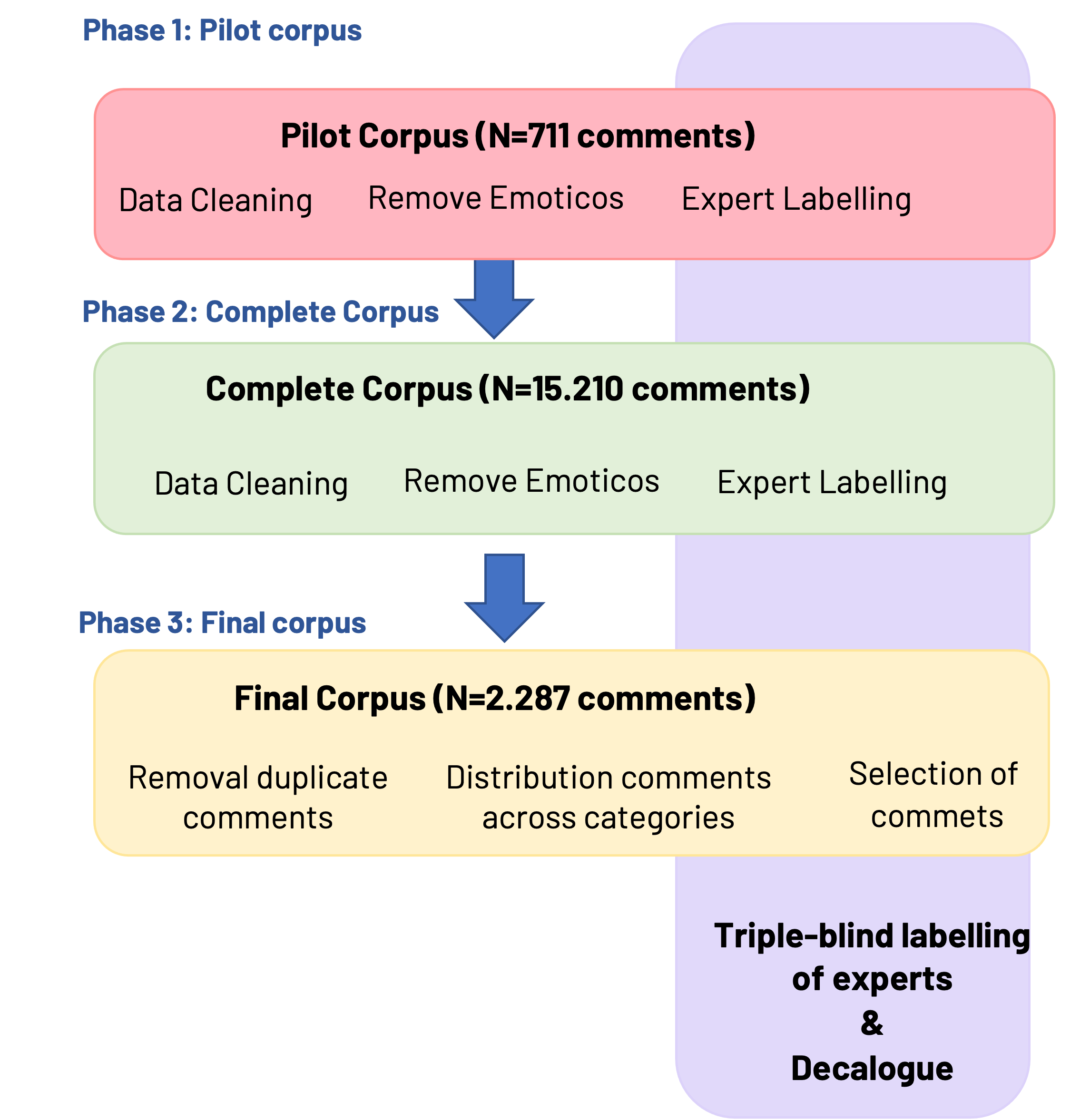

Supplement: Supplemental Information 13 [file peerj-cs-10-2251-s013.png]

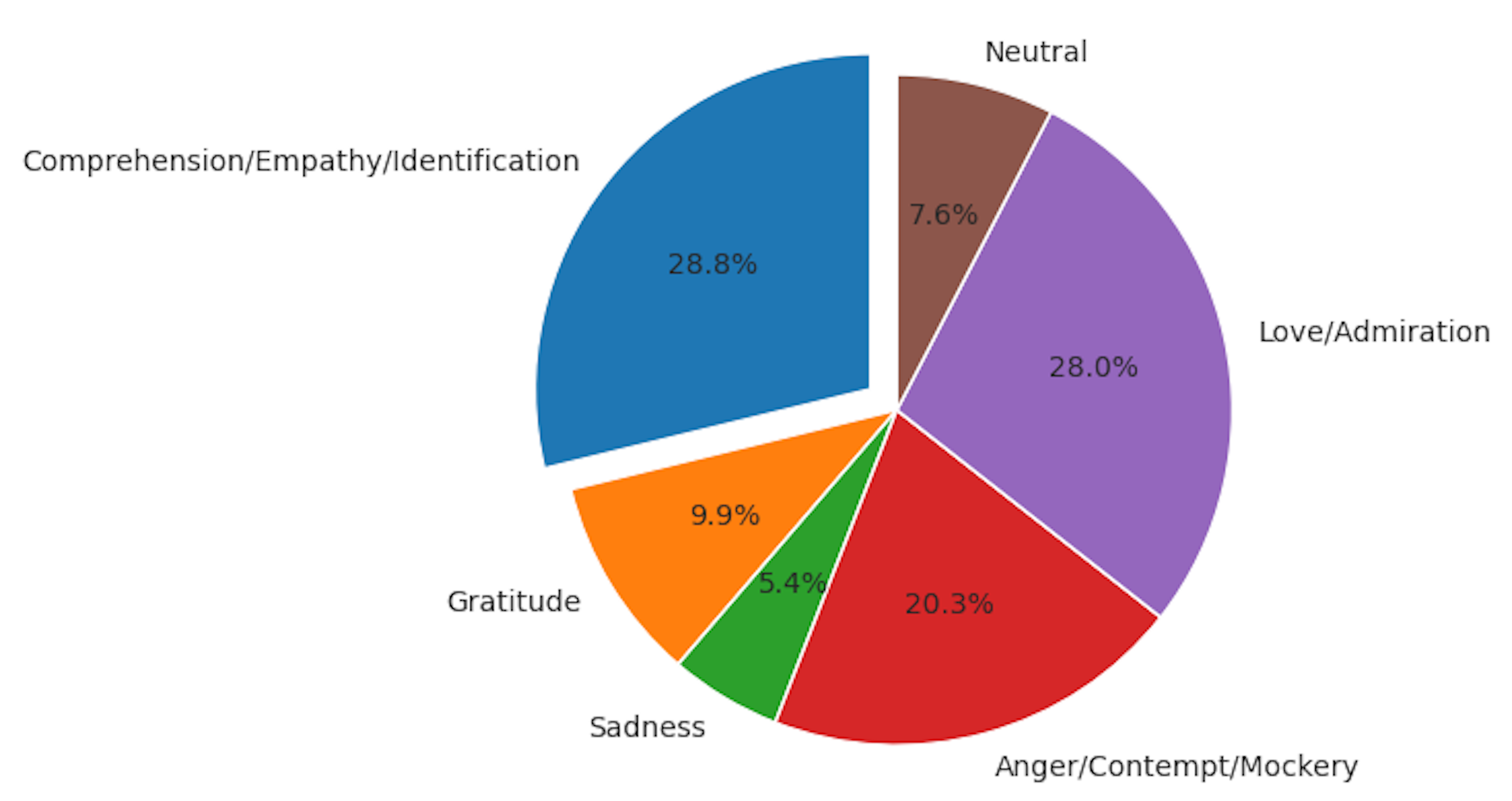

Supplement: Supplemental Information 14 [file peerj-cs-10-2251-s014.png]

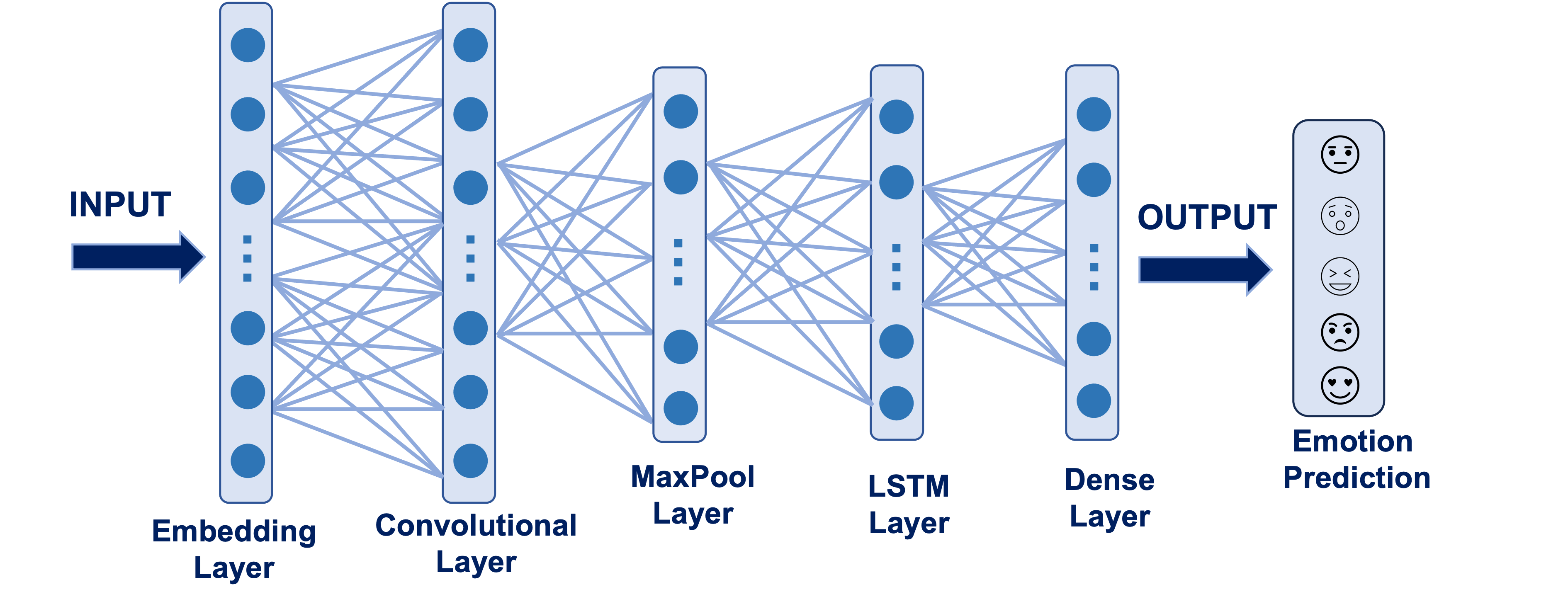

Supplement: Supplemental Information 15 — Own Elaboration [file peerj-cs-10-2251-s015.png]

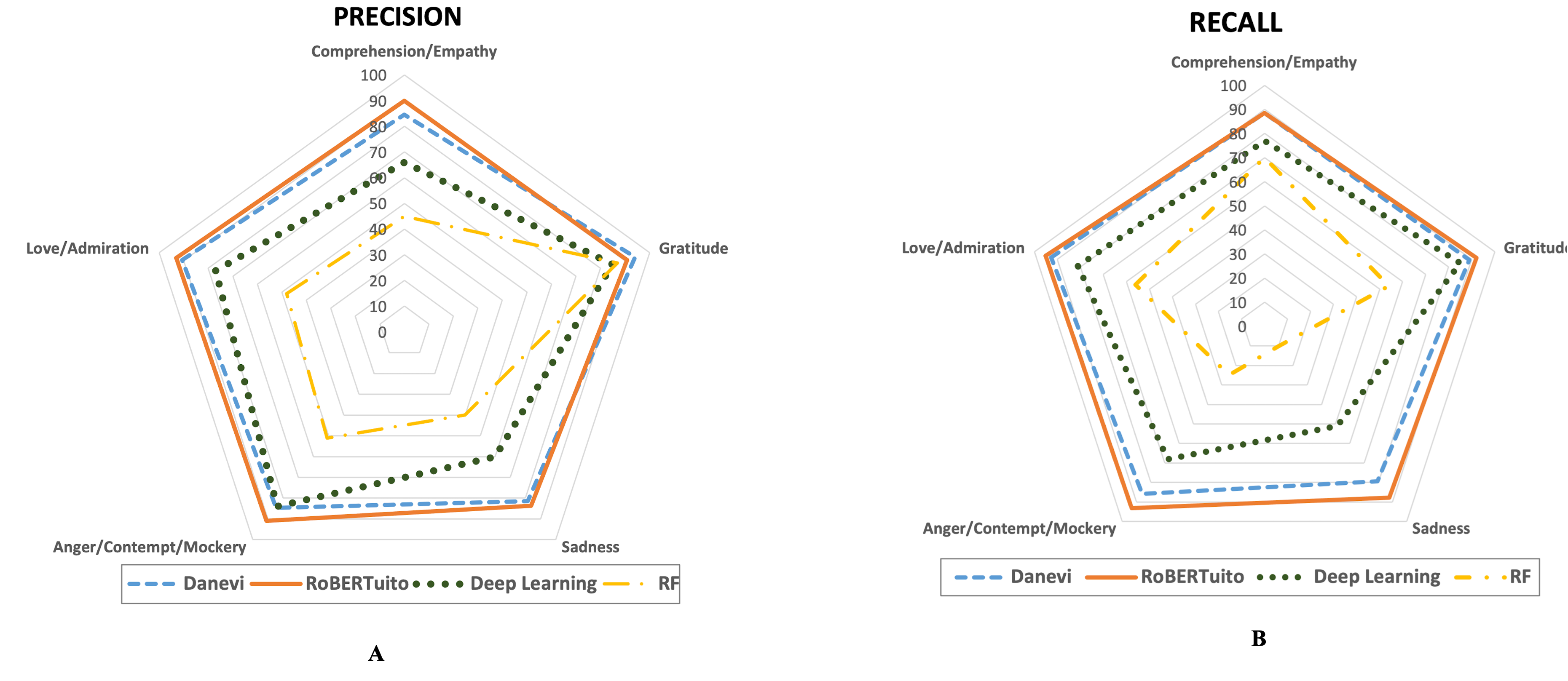

Supplement: Supplemental Information 16 — Own Elaboration [file peerj-cs-10-2251-s016.png]

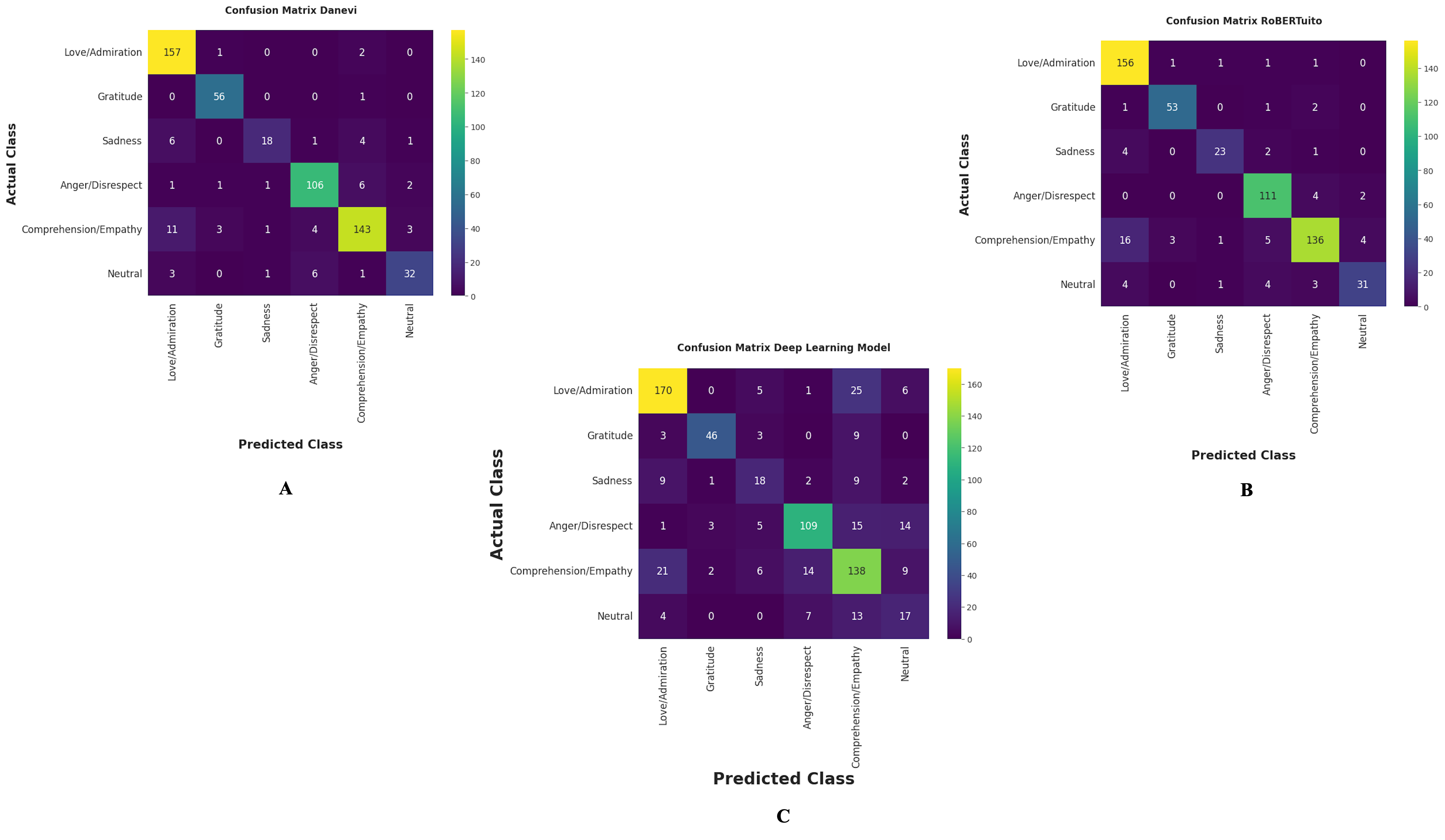

Supplement: Supplemental Information 17 — Own Elaboration [file peerj-cs-10-2251-s017.png]
